# The development of categorisation and conceptual thinking in early childhood: methods and limitations

**DOI:** 10.1186/s41155-020-00154-9

**Published:** 2020-07-22

**Authors:** Nicolás Alessandroni, Cintia Rodríguez

**Affiliations:** Department of Developmental and Educational Psychology, Universidad Autónoma de Madrid (Spain), C/ Iván Pavlov, 6, 28049 Madrid, Madrid Spain

**Keywords:** Concepts, Categorisation, Cognitive development, Early childhood, Research methodology

## Abstract

We present a systematic and qualitative review of academic literature on early conceptual development (0–24 months of age), with an emphasis on methodological aspects. The final sample of our review included 281 studies reported in 115 articles. The main aims of the article were four: first, to organise studies into sets according to methodological similarities and differences; second, to elaborate on the methodological procedures that characterise each set; third, to circumscribe the empirical indicators that different sets of studies consider as proof of the existence of concepts in early childhood; last, to identify methodological limitations and to propose possible ways to overcome them. We grouped the studies into five sets: *preference and habituation experiments*, *category extension tasks*, *object sorting tasks*, *sequential touching tasks* and *object examination tasks*. In the “Results” section, we review the core features of each set of studies. In the “Discussion” and “Conclusions” sections, we describe, for one thing, the most relevant methodological shortcomings. We end by arguing that a situated, semiotic and pragmatic perspective that emphasises the importance of ecological validity could open up new avenues of research to better understand the development of concepts in early childhood.

Concepts are commonly defined as representations of categories of things in the world, namely representations of groups of things that have something in common (Oakes 2008).^1^ As such, they are thought to enable us to inhabit the world in which we live by summarising its complexity, ordering it and adapting it to our limited cognitive capacities (Alvarez & Franconeri, 2007; Lakoff, 1990). By organising our experience into cognitive classes, concepts would act as mental stenographers that allow us, as Bruner, Goodnow and Austin (1956/2009, p. 1) say, to stop being *slaves of the particular*. Nevertheless, their importance does not end there. Concepts enable us to make inferences about the potential belonging of unknown entities to categories already formed (Medin & Abn, 2004). This two-way process (i.e., world summary ↔ categorical extension) is very convenient for the achievement of our practical objectives, as it is the basis for action planning and cognitive self-regulation (Clifton et al., 1991; Sakharov, 1990; Strauss, 1979; Zaporozhets, 2002). Without the degree of stability and certainty that concepts give us, we would not be able to direct our action in the world. And, indeed, without concepts that serve as referents of communication, we could not interact with others as we do (Roberts & Jacobs, 1991; Wright, 1913), which would condemn us to radical solipsism. Categorisation and conceptualisation are fundamental pillars of human cognition. Without them, it would not be possible to understand perception, memory, language or any form of general thought. They are “the glue that holds our mental world together” (Murphy, 2002, p. 1) and, so, stand as a matter of privileged study in cognitive sciences (Harnad, 2005).

Despite the unparalleled importance of concepts, not all authors share the idea that their origin can be traced back to the earliest moments of life (Poulin-Dubois & Pauen, 2017). After all, even the parents of developmental psychology placed the first manifestations of conceptual development beyond the frontiers of early childhood. Piaget, for example, assumed that *conceptual intelligence* does not develop until the period of concrete operations; for him, concepts need the classification and serialisation of reality through a taxonomic system of categories with fixed and arbitrary linguistic definitions (Piaget, 1951/1999, p. 220). For Vygotski, meanwhile, *genuine conceptual thinking* is a higher psychological function dependent on language that does not emerge until adolescence (Vygotsky, 1930/1998, p. 29). In his words, “real concepts are impossible without words, and thinking in concepts does not exist beyond verbal thinking” (Vygotsky, 1934/2008, p. 115). For Wallon, finally, concepts only emerge at the end of the long process of constitution of the cognitive object and presuppose both the passage from *situational intelligence* to *representational intelligence* and the development, around ten years of age, of a *classification rubric of reality* (Wallon & Ascoli, 1950; Wallon, 1942/1970). Thus, despite considering that cognitive development involves progressive constructions, when speaking of concepts these three authors refer to the mature and sophisticated forms of conceptual thought to which older children have access and not to their earliest potential origins.

The demanding threshold that Piaget, Vygotski and Wallon set for talking about concepts is due, in part, to their adherence to the Aristotelian perspective on concepts. According to it, concepts are nothing more than logical definitions consisting of sufficient and necessary sets of conditions that determine whether the entities of the world are enclosed in them (Smith & Medin, 1981). For decades, there have been critical voices arguing that the classical threshold to which we have referred is a theoretical obstacle rather than a good description of the development of cognitive skills (e.g., Fodor, 1972; Gratch, 1975; Starkey, 1981). As an example, Fodor has pointed out that Vygotskian theory converts child conceptual development into the gradual approach to the adult mastery of Boolean logic (Fodor, 1972, p. 90).

In opposition to classical positions in developmental psychology that relegate the emergence of genuine concepts to later ages, a number of studies have raised the possibility that categorical organisations and/or concepts exist from early childhood (e.g., Babska, 1965; Ricciuti, 1965; Ross, 1980; Schlesinger, 1982; Welch, 1939). In particular, since 1980 we have witnessed an explosion of studies on the origins of conceptual development in the 0-2 age period. These studies have not only argued that early conceptualisation is possible, but have also contributed to our conception of infants as psychological subjects who have at their disposal a rich and complex conceptual system. A noteworthy innovation is that these studies consider conceptual thinking to be expressed not only in linguistic expressions, but also in certain *concept-like behaviours* (see Bomba & Siqueland, 1983). By and large, these refer to cases where children exhibit a similar behaviour with different members of one category (e.g., several cups) or act differently with members of two or more categories. Nonetheless, depending on their theoretical and methodological framework, studies consider different behaviours as empirical indicators of conceptual thinking. Thus, for instance, behaviours such as looking at a certain stimulus or sequentially touching objects are considered, by some, as a window into the conceptual architecture of child cognition.

In this article, we present a systematic and qualitative review of academic literature on early conceptual development (0–24 months of age), with emphasis on methodological aspects. The main aims of the article were four: first, to analyse empirical studies and organise them into study sets according to methodological similarities and differences; second, to elaborate on the methodological procedures that characterise each study set; third, to circumscribe the empirical indicators that different study sets consider as proof of the existence of concepts in early childhood; last, to identify the most critical methodological limitations of research on early conceptual development and to propose possible ways to overcome them.

The analysis of the procedures used in each study allowed us to group them into five sets: *preference and habituation experiments*, *category extension tasks*, *object sorting tasks*, *sequential touching tasks* and *object examination tasks*. In the “Results” section, we review the core features of each set of studies and show consistencies and inconsistencies between empirical studies to assess the degree of congruence between them (e.g., if the different studies refer to the same thing when they speak of categories or concepts). In the “Discussion” and “Conclusions” sections, we examine, for one thing, methodological shortcomings. We further argue that a situated, semiotic and pragmatic perspective that emphasises the importance of ecological validity could open up new avenues of research to better understand the development of concepts in early childhood.

## Method

For the systematic review, we adopted the PRISMA declaration guidelines (Urrútia & Bonfill, 2010). We undertook a bibliographic query consulting ISI (Web of Science), Scopus, Proquest, ERIH, PubMed, ScienceDirect, PsycINFO, Psicodoc, Dialnet, SciELO and Latindex Catalog using the following keywords, in Spanish, English and French: *cognitive*, *development*, *concepts*, *categorisation*, *early childhood* and *child*. For the Boolean search, we combined keywords with the following operators: (cognitive AND development) AND/OR (“early childhood” OR child) AND (concepts AND/OR categorisation). This resulted in a total of between 306 and 52,104 records, depending on the language of the search, keyword combinations and the database consulted in each case. After checking for thematic appropriateness and removing duplicates, we ended up with a total of 1,601 records. Subsequently, we proceeded to the screening (considering titles and abstracts) and filtering (assessing full-text articles), according to the following eligibility criteria:

- *Publication type*: We only included empirical articles published in peer-reviewed scientific journals.
- *Publication area*: We only included articles reporting psychological research results.
- *Purpose of study*: We only included publications on conceptual and/or categorisation development in early childhood. Outcomes on related topics (e.g., the formation of memories, the development of perceptual discrimination, the development of object individuation, the development of associations between words and objects, and the development of phonetic discrimination) were excluded.
- *Age of the subjects involved in the studies*: Since our focus was early conceptual development, we only considered, as a rule, studies with participants up to 24 months of age. However, studies involving subjects older than 24 months were also considered if and only if they presented relevant and specific results for the age range considered in this review. For example, certain studies divide their total sample into age groups (with at least one of these groups being within the age range 0–24 months) and report different degrees of conceptual abilities for each of them (e.g., presence/absence of conceptual behaviours) (e.g., Babska, 1965; Bornstein & Arterberry, 2010; Sugarman, 1981). The chosen approach has the advantage of not leaving out potentially valuable empirical evidence.
- *Language*: We only included publications written in Spanish, English or French.
- *Duplicates*: The results of a same empirical investigation that were reported more than once (i.e., in two or more different articles) were excluded.
- *Replica experiments*. A considerable number of them presented the results of replica experiments to which other articles referred. To avoid basing our methodological comparison on the number of replicas of certain methods rather than on the nature of those methods (which would have been a bias for a methodological review), replica studies were excluded.

**Fig. 1:**
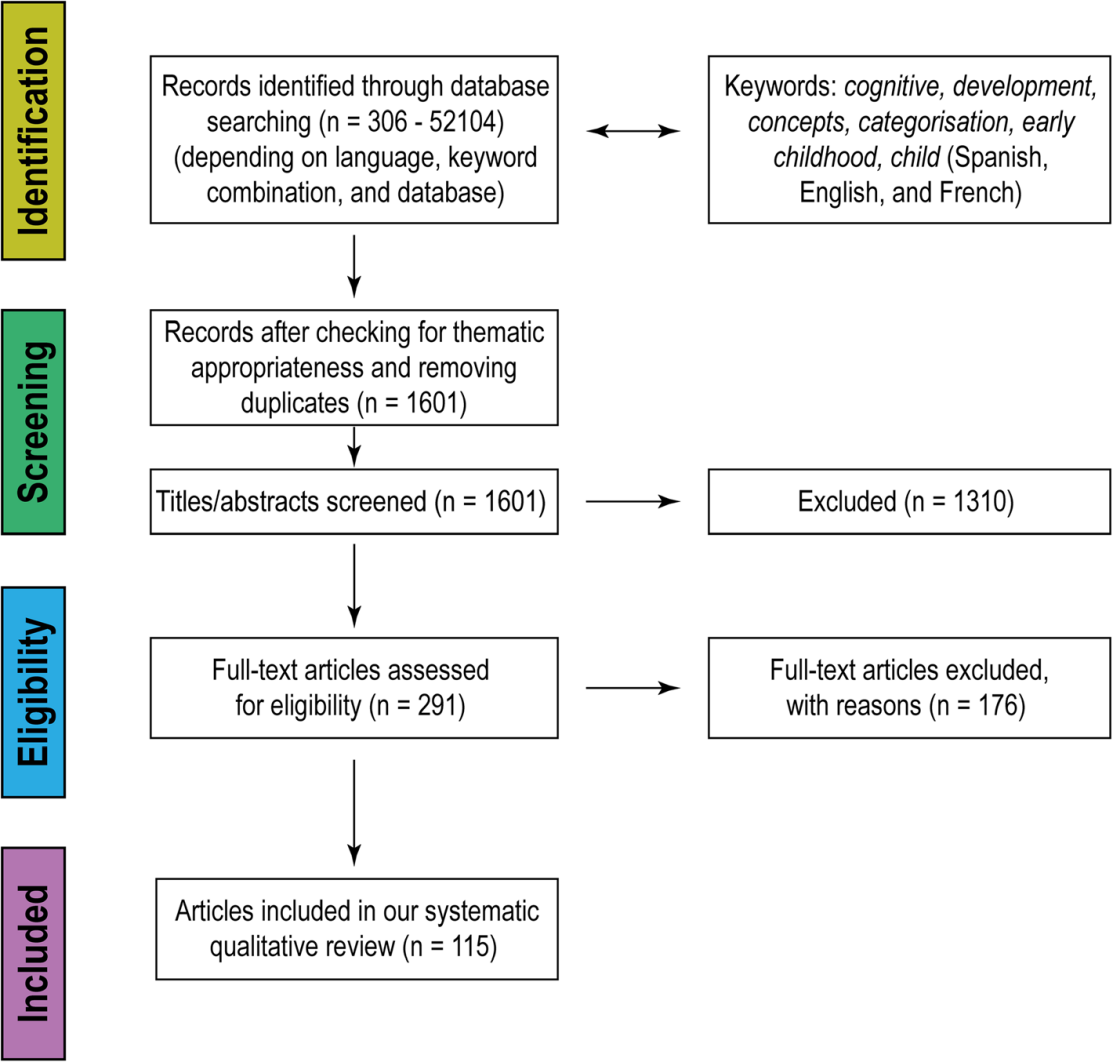
PRISMA flow diagram

The final sample consisted of 115 articles reporting 281 studies (see Fig. 1).

**Fig. 2:**
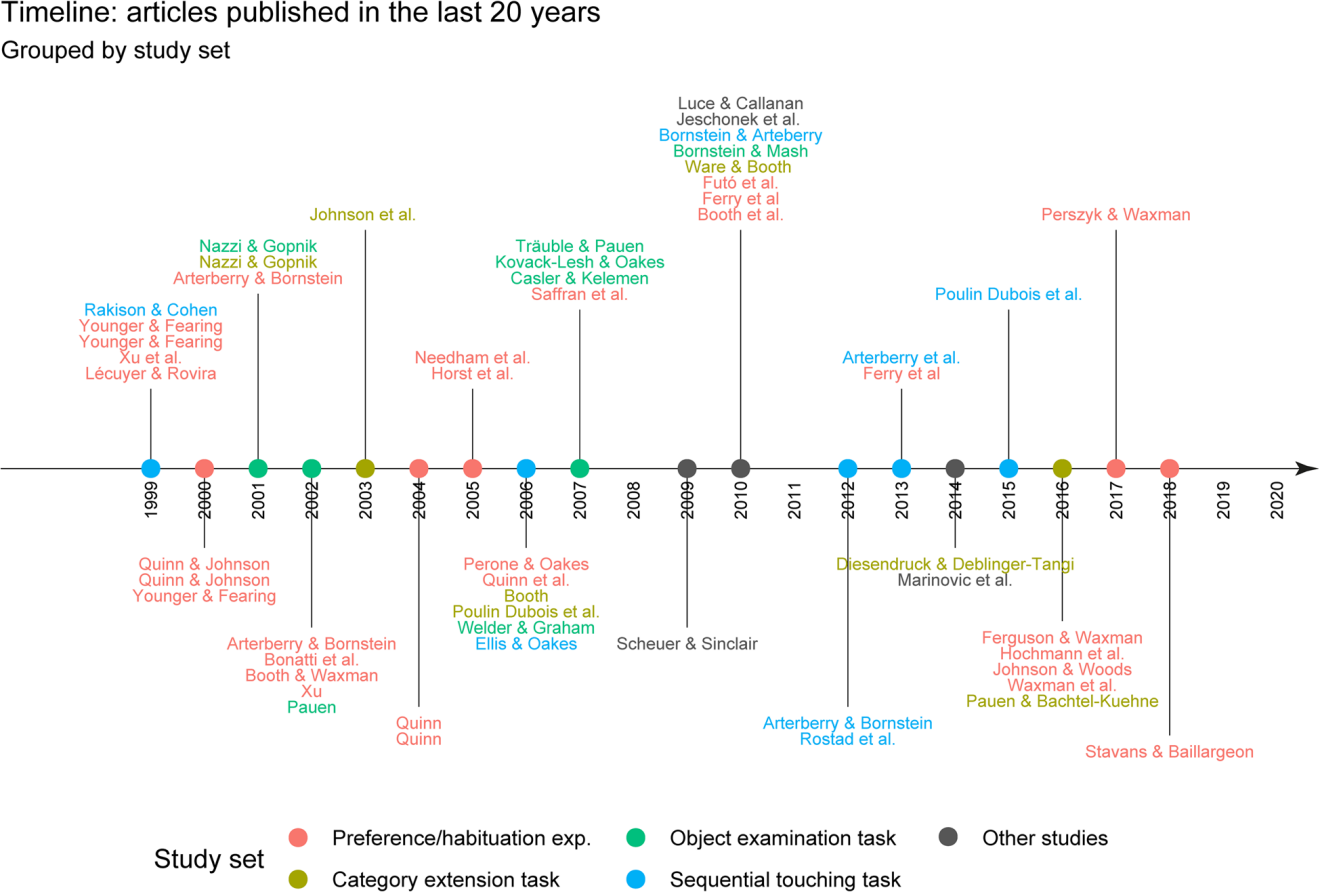
Timeline: articles published in the last 20 years. Grouped by study set

For each study, we analysed the dimensions listed in Table 1. The procedural comparison gave rise to five emerging sets to classify the studies: *preference and habituation experiments*, *category extension tasks*, *object sorting tasks*, *sequential touching tasks* and *object examination tasks*. We also included a set of *other studies* not congruent with the other emerging sets. Table 2 summarises the frequencies for each set and their breakdown according to the design type, its temporality, the sample type and the kind of stimuli used in each case. Since our review did not consider the year of publication as an eligibility criterion, we provide, in Fig. 2, a timeline showing articles published in the last 20 years (*n* = 50), disaggregated according to the set of studies to which the studies they report (*n* = 54) belong.

**Table 1:** Analytical dimensions for the review

A. Year of publication	
B. Research design 1. Descriptive 2. Experimental	
C. Sample size	
D. Subsample size (studies with two or more experimental conditions)	
E. Sample/subsample type. Based on sample/subsample sizes. 1. Case study: one subject 2. Intensive: up to 30 subjects 3. Extensive: more than 30 subjects	
F. Temporality 1. Cross-sectional 2. Cross-sequential 3. Longitudinal	
G. Age range of subjects (in months)	
H. Stimuli 1. Two-dimensional i. Drawings and images ii. Abstract patterns iii. Videos 2. Three-dimensional i. Objects: material entities that have not been built to fulfil a particular purpose but can be easily recognised by members of a community (e.g., a piece of mass, geometric shapes of various materials) ii. Artefacts: material entities constructed by a certain technique to fulfil a culturally determined canonical purpose (e.g., a spoon or a ballpoint pen). iii. Object, artefact and living beings replicas: material entities (usually small) that exactly reproduce the characteristics of an object/artefact or living being (e.g., a toy horse, a toy spoon). iv. Non-objects: material entities that resemble an object or artefact but would not be accepted as such by members of a community (e.g., stimuli constructed by experimenters manipulating different dimensions of materiality). 3. Language 4. Sounds and music	
I. Data analysis 1. Quantitative 2. Qualitative 3. Mixed	
J. Age of development of categorical representations (in months)

**Table 2:** Number of studies for each set in relation to design type, temporality, sample type and stimuli

Study set	Design type		Temporality			Sample type			Stimuli^+^								
	*D*	*E*	*Cs*	*Cq*	*L*	*CS*	*Int*	*Ext*	*R*	*O*	*A*	*NO*	*D/I*	*P*	*V*	*L*	*S/M*
Preference and habituation	-	156	141	15	-	-	153	3	18	18	6	5	86	15	17	11	12
Category extension	1	48	37	10	2	-	46	3	12	8	18	13	3	-	3	2	-
Object sorting	-	5	3	2		-	5	-	2	3	-	3	-	-	-	2	-
Sequential touching	-	27	7	18	2	-	25	2	28	5	4	1	2	1	-	-	-
Object examining	-	34	22	11	1	-	32	2	21	2	-	13	-	-	-	4	-
Other methods	7	3	3	3	4	2	8	-	1	1	3	-	2	-	-	-	-
**Total**	8	273	213	59	9	2	269	10	82	37	31	35	93	16	20	19	12

*Note*. D= descriptive, E= experimental; Cs= cross-sectional, Cq= cross-sequential, L =longitudinal; Cs= case study; Int= intensive, Ext= extensive; R= replicas, O= objects; A= artefacts; NO= non-objects; D/I= drawings and images; P= patterns; V= videos; L= language; S/M= sounds and music. ^+^ As a single study can feature more than one type of stimuli, the sum of the number of studies for each column does not reflect the total number of studies we analysed

## Results

### Preference and habituation experiments

Preference and habituation experiments are two predominant approaches in psychological research. Originally used to assess the existence of simple sensory discriminations and preferences (e.g., Valentine, 1914), these procedures soon extended to the study of attention to sensory patterns (e.g., Fantz, 1964; Fagan, 1976), the memoristic retention of incidental visual experiences (e.g., Fagan, 1970) and object permanence (e.g., Baillargeon et al., 1985), among other matters.

Generically, researchers use procedures with a high level of control (i.e., in laboratory settings) to assess whether there are differences in the fixation of gaze to two stimuli or groups of stimuli of which only one has been presented to children in a preliminary phase of familiarisation. The underlying assumption is that babies’ gazes are not random, but rather reflect cognitive processes accounting for children’s exploratory behaviours and orientation responses (Berlyne, 1958) or sophisticated statistical inferences (Sim & Xu, 2019). Since children have a natural propensity to direct their gaze to stimuli from the first months of life (Weizmann et al., 1971), this method has been used to study cognitive development from very early ages (from 8 weeks of age; for a discussion, see Olson, 1976).

When there are statistically significant attentional biases, researchers interpret them as indicating cognitive processes. In the field of early conceptual development, this general procedure takes on certain particularities.

In the *familiarisation phase*, subjects are habituated with stimuli that the researchers consider as exemplars of the category for which they seek to assess whether children have a conceptual representation (e.g., animals, fruits or geometric shapes). This is done by means of different tests whose duration is, for the most part, relatively short (i.e., of the order of a few seconds). Stimuli are quite varied: in our review, we found studies using images, drawings, abstract patterns, objects, artefacts, replicas, non-objects, videos, language, sounds and music.

Familiarisation trials can be of two types: *fixed* or *infant-controlled* (Colombo & Mitchell, 2009). When trials are fixed, both their duration and the duration of inter-trial intervals are predetermined. In this case, stimulus presentations are not linked to infant behaviour: babies may or may not watch the stimulus without affecting the development of the procedure. Infant-controlled trials, on the contrary, begin when children look at the stimuli and end when children look at something different. Also, these trials continue until the measure of infant attention has met a predetermined criterion (e.g., it has decreased by a certain percentage).

During the *test phase*, children are introduced to new exemplars of the category with which they have previously become familiar, as well as exemplars of a new category. The test stimuli may be presented concurrently (i.e., one familiar stimulus vs. one novel stimulus, all at once) or sequentially (i.e., one familiar stimulus followed by one novel stimulus). The calculation of gaze fixation times for each kind of test stimulus results, then, in a *preference score*, which typically expresses the quotient between the baby’s gaze fixation time to the exemplars of the novel category and the total gaze fixation time (i.e., to exemplars of both the familiar and novel categories).

When this score is statistically significant, researchers establish that the baby has extended the generalisation of habituation to new exemplars of the familiar category (but not to exemplars of the novel category) and report the existence of concept-like behaviours (Bomba & Siqueland, 1983). In other words, the differential attention to exemplars of the novel category is explained arguing that children recognise these exemplars as belonging to a different class than the one containing the familiar exemplars—despite the differences that may exist between the latter (Quinn et al., 2006). Finally, the procedure is usually complemented by using the paradigm of preferential looking as an experimental control, both to assess whether subjects are capable of distinguishing members of the same category from each other and to rule out the existence of a priori inter-categorical preferences (e.g., Eimas & Quinn, 1994, exp. 3 and 5; Quinn & Eimas, 1996, exp. 3, 5 and 6; Quinn & Johnson, 2000, exp. 3; Younger & Fearing, 1999, exp. 3).

At the theoretical level, it is assumed that differences in gaze fixation prove the existence of a children’s capacity to make judgments of relative similarity based on information encoded during the familiarisation phase (Sherman, 1985) regarding the perceptual characteristics of stimuli (i.e., shape, colour, movement) (Quinn et al., 1993). In this manner, during familiarisation, subjects would form a mental representation that “allows the familiarity attached to the previously encountered category instances to generalise to novel instances of the familiar category” (Quinn & Eimas, 1996, p. 190).

Through this method, for example, Quinn and Johnson (2000) habituated 2-month-olds with images of different cats. In the first test phase, they simultaneously presented images of a new cat and a rabbit or an elephant. In the second test phase, they presented images of a new cat and a dog. The data revealed that there were no statistically significant differences in preference scores and that, therefore, babies did not form a basic-level representation for cats that excluded exemplars of other animal species. Yet, when performing the procedure at another level of abstraction (global level), the results were different. Another group of children of the same age was habituated with images of mammals. After the test phases (new familiar mammal vs. unknown mammal; unknown mammal vs. furniture exemplar), the researchers found that at 2 months children can form a global representation of mammals that includes exemplars of mammals not seen during familiarisation but excludes furniture. Along the same lines, Arterberry and Bornstein (2001, 2002) habituated 3-month-old infants with static images or dynamic dot patterns from the same set of animals and vehicles. Subjects presented a significant preference for exemplars of novel categories in both habituation conditions, demonstrating that infant categorisation of animals and vehicles makes use of both static and dynamic information.

These results complement those of other familiarisation studies with images concluding that, from 3 months of age, children are capable of forming basic-level categories of animals and furniture (Behl-Chadha, 1996; Eimas & Quinn, 1994; Quinn et al., 1993; Quinn & Eimas, 1996, see also Roberts, 1988 with 9-month-olds; Younger & Fearing, 1999, 2000 with 10-month-old children), geometrical shapes, and patterns of sequenced stimuli (Bomba & Siqueland, 1983, see also Saffran et al., 2007 with 7-month-olds; Colombo et al., 1990 with 10-month-old infants; Hochmann et al., 2016 with 14-month-olds). There is also evidence that accompanying habituation with images with words or vocalisations of non-human primates—but not other sounds—favours the categorisation of objects in 3-month-old babies (Ferry et al., 2010, 2013; Perszyk & Waxman, 2017; see also Balaban & Waxman, 1997 with 9-month-old children). Moreover, studies show that from the age of 6 months children exposed to communicative tones show an advantage in the categorisation of objects (Ferguson & Waxman, 2016) and that children from 7 months have categorical representations of female faces (Cohen & Strauss, 1979).

Habituation experiments have also involved three-dimensional stimuli: replicas, objects, artefacts and non-objects. For example, Needham et al. (2005) habituated 4-month-old infants with boxes of the same size and shape, but of different background colour, detail colour and detail shape. In the test phase, babies saw a different box next to a cylinder. In one experimental condition, the box and cylinder moved as a unit. In a second experimental condition, the movement of the box was independent of that of the cylinder. When they familiarised children with three boxes of different background/detail colour and the box used in the test phase was like the one used during habituation, children fixed their gaze more during the first experimental condition. The authors explain this effect by arguing that, under such conditions, subjects can form a conceptual category that allows them to differentiate cylinders from boxes. For his part, Xu (1998, 2002) verified that, at 9 months of age, if visual familiarisation with two stimuli (a toy duck and a ball; a cup and a shoe for babies; a replica animal and a non-object constructed for the experiment) is accompanied by two linguistic labels—one per stimulus, the differential categorisation of the stimuli is facilitated. This effect was not maintained when habituating children using a single linguistic label, two tones, two sounds or two emotional expressions.

Other studies with three-dimensional stimuli suggest that from 9 months infants have representations of shape-based object categories (Ruff, 1978; see also Ross, 1980, with children aged 12–24 months) and colour-based object categories (Johnson & Woods, 2016), that 12-month-olds use kind information to individuate objects (Xu et al., 1999), and that at 14 months children can form a contextual category of “kitchen items” (e.g., an apple, a high chair, a bib, a sponge, a carton of milk, a banana, an oven and a refrigerator) that excludes “toiletries” (e.g., a bathtub, a toothbrush, a towel, toilet paper, a Vaseline container, a brush and a packet of disposable tissues) (Mandler et al., 1987, exp. 1). Finally, some authors propose that, from 4 months, knowledge about object functions facilitates the categorical representation of artefacts (Stavans & Baillargeon, 2018; see Futó et al., 2010; Horst et al., 2005 with 10-month-olds; Booth & Waxman, 2002; Booth et al., 2010 with 14-month-old children). This evidence supports the possibility that first concepts are not motivated solely by perceptual similarities.

### Category extension tasks

Early conceptual development has also been researched through *category extension tasks*. Those who have used these tasks often argue that it is not enough to assess the vicissitudes of children's visual attention, but that other contextual factors must also be considered. For instance, Babska (1965) has argued that it is necessary to consider the conditions under which cognition finds its expression in a *form of activity*. This interest in the deployment of action in specific contexts has determined that most of these tasks employ three-dimensional stimuli with which children can act (replicas, objects, artefacts and non-objects). However, these tasks should not be confused with explorations in ecological contexts, as here researchers tend to manipulate and control variables.

Typically, category extension tasks involve a first *phase of training or behavioural conditioning* with a repeated stimulus or with several exemplars of a class. Once a homogeneous response has been established or learned, the researchers proceed to the *generalisation phase*, in which children are presented with other stimuli. These typically include variations of the stimuli presented during the training phase. If children extend the learnt behavioural pattern to the new stimuli, it is concluded that they understand that the stimuli presented during both phases belong to the same category. This would be possible thanks to the codification of a general representation during the training phase. This representation would allow children to ignore the categorically irrelevant differences between the stimuli of both phases.

For example, Welch (1939) conditioned two boys and two girls between 18 and 20 months of age by inducing them to take an 8-inch square plywood side plate while being told the word “ate”. During the generalisation trials, children were taken to a room with four or five objects. One of these objects was a plate nearly identical to the training plate, but with different dimensions (15.75 × 0.15 inches). Subjects were then asked to take the “ate”. When they failed, a new test was performed with a plate whose dimensions were more similar to those of the training plate. Each positive response (i.e., taking the new plate) was interpreted as an indication of the existence of a categorical representation of “plate” resistant to size modifications. Similarly, Ross et al. (1986) showed that at 20 months children can extend arbitrary names learned when seeing four groups of objects to new instances of those groups. Other studies, meanwhile, found generalisation answers regarding other object properties, such as shape or colour (e.g., Hayne et al., 1987, at 3 months; Clifton et al., 1991, at 6 months; Collard & Rydberg, 1972, at 8 months) or canonical orientation (Freeman et al., 1980, at 9 months).

In another classic study, Fagen et al. (1976) trained 3-month-old children by placing a five-component mobile with animal figures over their cribs. They placed a ribbon connecting a baby’s ankle to the suspension bar from which the mobile hung, so that babies could establish an association between their body movement and the movement of the mobile (see Rovee & Fagen, 1976 for a detailed description of the methodology). When, 24 hours later, they switched from the original mobile to other mobiles exhibiting zero to four differences (i.e., new components), they observed that subjects performed kicks when one or two elements were changed, but not when three or four were changed. This result was interpreted as evidence in favour of the retention of the stimulus-response interaction in the medium and long term based on the codification of a categorical representation, both in this study and in an extension of it carried out with 3-month-olds (Greco et al., 1990).

A third form of category extension task can be found in studies addressing children’s *inductive generalisation* and its relationship to concept formation (Mandler, 2003; Mandler & McDonough, 1996, 1998; McDonough & Mandler, 1998). We refer to studies where the training phase stimuli are different from those of the generalisation phase. The variety of stimuli evaluates whether children generalise properties from one conceptual domain to another and not only from one object to another case of the same object (e.g., Baldwin et al., 1993). The standard procedure is simple: first, researchers repeatedly demonstrate to children an action with a modelling exemplar and a prop, accompanying this demonstration with linguistic information. This procedure is called *action modelling* and involves the training of a behavioural response. Then, two new modelling exemplars are placed in front of the children at the same time. One of them is a new exemplar of the same category as the main training object. The other object acts as a distractor (i.e., belongs to another category). Finally, the prop with which the child has been trained is handed over, and the linguistic information is provided again. Researchers then evaluate whether children extend the learned action to the object belonging to the same category as the modelling exemplar or to the new distracting object. Following this methodology, Mandler and McDonough (1996) found that, at 14 months, children who were shown the actions of “drinking from a glass” and “lying on a bed” with a replica dog, while being told “sip, sip, umm, good” and “night night”, respectively, generalised these actions to other animals, but not to vehicles. Conversely, after modelling with a replica car, children generalised the properties of “owning a key” and “taking a ride” to other vehicles, but not to animals. In one replica (Mandler & McDonough, 1998), the authors found the same results with 9-month-olds, suggesting that, from that age, babies have global concepts of animals and vehicles.

Other research employing these and other versions of category extension tasks provided additional evidence of the generalisation of body, sensory and movement properties with animals and people (Diesendruck & Deblinger-Tangi, 2014 at 19 months; Poulin-Dubois et al., 2006 at 14 months), functional properties with objects (Booth, 2006 at 18 months; Pauen & Bechtel-Kuehne, 2016 at 18 months; Ware & Booth, 2010 at 19 months) and movement trajectories with abstract two-dimensional stimuli (Johnson et al., 2003 at four months).

### Object sorting tasks

We have mentioned earlier that, for certain authors (Piaget, 1959/1991; Vygotsky, 1934/2008), true concepts are developed in adolescence, when subjects come to fully understand the logical and taxonomic relationship that exists between a category and a subcategory as a consequence of the development of formal operations or higher psychological functions. The lack of this understanding would be responsible, from this perspective, for the failures and limitations that children exhibit in classic object categorisation tasks.

In a pioneering work, Ricciuti (1965) proposed that the “failure” of subjects in classical tasks (e.g., the inability of children to verbally report why they had acted in a certain way or the vagueness of the meaning of a word) was not the product of an incomplete conceptual development, but of the difficulty that the complicated linguistic instructions of those tasks posed to them (see also Ricciuti & Benjamin, 1957). This author also emphasised the importance of considering the behavioural responses that children give outside the semiotic system of language. Specifically, he proposed that despite not being able to follow verbal instructions, many children do group objects systematically and spontaneously and that this is a sufficiently valid indicator to study children’s conceptual formation. This proposal thus links *object manipulation* with alleged *categorical and/or conceptual behaviours*, that is, organised behaviours of a general type, based on cognitive rules (Nelson, 1973). Following this hypothesis, different authors designed more straightforward *object sorting tasks* to evaluate the behaviours deployed by children during tasks. The underlying premise of these tasks is that if children have cognitively represented classes (i.e., concepts), they will form groups of objects by separating in space those belonging to the same category from those belonging to another.

Ricciuti (1965), for example, conducted an experimental study with 48 children aged 12, 18 and 24 months who were involved in four simple object sorting tasks. In each task, he presented the children with a tray with eight mixed objects (e.g., cubes, spheres and polygons), four from one class and four from another. The classes differed in colour, size and/or shape of the objects involved. He then said to each child to “you play with them—you fix them all up” (Ricciuti, 1965, p. 132), for which he allocated a period of up to two and a half minutes. The results of the experiments showed that, from the age of 12 months, some children manipulate objects belonging to the same class in successive ways and form spatially differentiated groups with objects belonging to the same class. In other words, since the age of 12 months, some children seem to engage in categorical behaviours that could be considered preverbal instances of conceptual formation.

These results have been endorsed by research with both younger and older subjects. Starkey (1981) investigated the ability of 6-, 9- and 12-month-old babies to form groups with objects different in size, colour and/or shape. His results showed that although 6-month-olds are not able to group objects (probably due to a lack of development in their motor skills), since the age of 9 months, babies exhibit certain rudimentary grouping behaviours that, at 12 months, show a higher degree of organisation. For its part, Nelson (1973) found that at 19 months of age children form groups of objects based on differences in the dimensions of colour, shape and function of objects (i.e., any action associated with objects), both independently and in combination. These results are consistent with the findings of Corrigan and Schommer (1984), which show that at 24 months children presented with non-objects with different form but similar function (and vice versa) in situations of social interaction with adults group objects according to both their physical and functional attributes.

### Sequential touching tasks

As mentioned, Ricciuti (1965) stressed the limited scope of the classical tasks of object categorisation and proposed to consider new indicators of early conceptual development (i.e., the spatial grouping of objects). Similarly, Sugarman (1981) argued that grouping tasks are restricted and can be extended. According to her, a complete analysis of categorical behaviour should not only consider object grouping behaviours but also *object selection behaviours*. In other words, children would not only manifest conceptual consistency by spatially organising objects, but also by selecting them (i.e., touching them) in a given order. This perspective gave rise to *sequential touching tasks* that, as the name implies, assess the extent to which children sequentially touch objects belonging to the same class. When this occurs to a higher degree than could be expected by chance, the subjects’ behaviour is explained positing the existence of categorical representations (i.e., concepts).

In one study, Sugarman (1981) investigated 40 children at 12, 18, 24 and 30 months of age, presenting them with seven sets of objects. Each set contained eight objects, of which four belonged to one category and four to another, different in shape and/or colour. Due to the lack of a tradition in studies using sequential touching tasks, Sugarman suggested an innovative data analysis that contemplated, on the one hand, the *overall ordering tendency*. She included in this measure (i) the relationship between two objects selected in succession in terms of their belonging to a class (i.e., same/different category) and (ii) the percentage of selected objects of each class, for each set. In addition, she took into account the presence of *extended ordering sequences*, a measure that considered (i) concatenated selections of specific exemplars of the same class or of different classes (such as *doll*_*1*_*- > doll*_*2*_*- > doll*_*1*_) and (ii) the number of sequences involving three and four members of the same class. Her results confirmed the existence of a statistically significant tendency to touch, in sequential order, objects belonging to the same class at all ages and a progressive increment, for each age, in the number and length of extended selection sequences.

Mandler et al. (1987) also used this procedure. Children aged 14 and 20 months were presented with three-dimensional replicas of “animals” versus “cars” and “bathroom objects” versus “kitchen objects”. After being asked “What can you do with all these things?” they let the subjects manipulate the objects for 2 min, without giving them any feedback. The authors’ analysis considered the *mean lengths of the runs* of successive touches from a category and compared them to the expected *chance run length* (see Mandler et al., 1987, p. 348). Although not statistically significant, the results showed children’s sensitivity to all categories at both ages. They also found that while at 14 months more children are *simple categorisers* (i.e., they systematically touch objects of a single category), at 20 months the ratio between these and *exhaustive categorisers* (i.e., they systematically touch objects of both contrasted categories) is equal.

Thanks to sequential touching tasks, there is a large body of evidence that concepts exist in early childhood. For example, Mandler and Bauer (1988) reported that from 12 months boys and girls perform sequential touches based on basic (e.g., dogs and cats), superordinate (e.g., animals and vehicles) and contextual (e.g., kitchen objects, bathroom objects) categories (see also Poulin-Dubois et al., 2015). Mandler et al. (1991), in turn, argued that at 18 months subjects have the global categories of “animal”, “vehicle”, “plant”, “furniture” and “kitchen utensil”, but show strong difficulties in differentiating the basic-level categories contained in these domains. This achievement would only become accessible at 30 months of age. The idea that global categories are the first to be formed is also endorsed by Bornstein and Arterberry (2010), who found that children between 12 and 30 months of age first form more inclusive level categories in the domains of animals, vehicles, fruits and furniture.

From another line of research, Rakison and Butterworth (1998a, 1998b, see also Rakison & Cohen, 1999) studied the categorisation of animals, insects, vehicles and furniture in children aged 14 to 22 months by introducing a new sub-type of the procedure, the *confusion task*. The novelty is that this task modifies the structure of parts of objects that work as stimuli (i.e., parts are removed, shifted or added) to assess whether children categorise by looking at the object as a whole (taxonomically) or specific parts of the object (“partonomically”) (see Tversky & Hemenway, 1984). Evidence showed that children use information about specific parts of objects to classify. Thus, for instance, they formed two different categories when exposed to animals with legs and to vehicles with wheels, but not when all replicas had the same parts (i.e., legs or wheels) or did not have common salient parts (i.e., did not have legs or wheels). Also, authors found that these findings hold up when introducing replicas with parts in unusual orientations, but not when parts violate the usual global configuration of objects, suggesting that from 14 months subjects treat objects as totalities and, at the same time, as collections of parts.

Finally, other works demonstrated that 14-month-old children can flexibly categorise a collection of objects according to their shape or the characteristics of their material (Ellis & Oakes, 2006). In addition, at this age, children can differentiate between brushes and telephones—but not pears and lemons—both with artefacts and three-dimensional replicas (Arterberry & Bornstein, 2012) and have already developed the animate/inanimate conceptual distinction (Rostad et al., 2012).

### Object examination tasks

The last group of studies corresponds to object examination tasks. The origin of this procedure can be traced back to the contributions of Ruff (1986), who demonstrated that the time children between 7 and 12 months spend examining an object (i.e., looking at it closely or inspecting it manually) is inversely proportional to the degree of familiarity they have with the object, something that also happens with gaze in habituation studies. Based on this, the author proposed that object examination reflects attentional cognitive processes and the incorporation of environmental information. Relying on Ruff’s contributions, Oakes et al. (1991) proposed to study early conceptual formation combining the logic of the habituation paradigm with object examination as a dependent variable. They claimed that this methodology allows for solving some problems of the other approaches. For example, before the age of 12 months, performance in sequential touching tasks could be subsidiary to children’s motor skills and not to their conceptual development. Besides, in sequential touching tasks, subjects are not required to respond to all stimuli (i.e., they may ignore some of them). Object examination tasks, on the other hand, pose minimal motor demands (since looking in focus is considered a form of examination) and require a response of the subject to each object. Also, object examination tasks are more ecologically valid than traditional habituation experiments because they involve a higher level of activity on the part of the subject.

In a *familiarisation phase*, children are habituated to objects of the same category (e.g., “vehicles”) which are repeatedly placed within their reach in different trials. Subsequently, in the *test phase*, researchers present a new object of the category that is allegedly known and a new object belonging to another category, one at a time. After comparing the examination times for each of the test stimuli, “if infants increase examining of novel toys from a novel category, but not to novel toys from the familiar category, then it is clear that they have responded in terms of category membership” (Oakes et al., 1991, pp. 380–381).

Following this procedure, Bornstein and Mash (2010) showed that at 5 months children can distinguish between two categories of non-objects that are different in colour and shape, both with pre-task training (i.e., seeing at home images of stimuli 2 months before the test) and without any training. For their part, Oakes et al. (1991) verified the existence of a conceptual distinction between trucks and animals from 6 months of age, congruent with the results of Mandler and McDonough (1993), who reported that from 7 months of age there are conceptual categorisation processes in the domains of animals and vehicles. In addition, they showed evidence that, by 9 months, subjects distinguish, at the basic level, between cars, motorcycles and aeroplanes (see also Pauen, 2002a). Pauen (2002b), meanwhile, showed that 10-month-olds categorically distinguish replicas of animals and furniture, even when there are inter-categorical perceptual similarities (see also Kovack-Lesh & Oakes, 2007 for a study on the categorisation of horses versus dogs). This suggests that there may be other sources of information to form categories, such as object functions (see Träuble & Pauen, 2007).

Evidence from studies with children aged 1 to 2 years is consonant with the results of studies with younger subjects. On the one hand, it confirms categorical sensitivity to non-objects with different functions (e.g., Madole et al., 1993; see also Casler & Kelemen, 2007) or physical characteristics (e.g., Welder & Graham, 2006). On the other hand, it shows that this sensitivity is favoured if nouns or adjectives are proffered when presenting objects (Nazzi & Gopnik, 2001; Waxman & Markow, 1995).

### Other procedures

Ten studies could not be classified into any of the sets described above. As they do not show unity at the procedural level, we chose instead to mention some of them briefly in this section. For one thing, we have included here two neuropsychological studies that measured the brain activity of infants between 4 and 8 months when they were shown random images of exemplars from the “animate/inanimate” (Jeschonek et al., 2010) or “human/animal” (Marinović et al., 2014) global domains. The studies showed that from 4 months, there are differences in neural responses to stimuli, which the authors interpreted as evidence of categorical brain processing.

There are also descriptive studies carried out in ecological contexts without variable control. These studies, based on observation, meticulously describe conceptual behaviours. Connolly and Dalgleish (1989), for instance, reported the longitudinal evolution of the ability to use a spoon by its function during the second year of life. They identified changes taking place in dimensions such as gripping strategies, visual monitoring and contralateral hand activities. The authors’ interpretation is that between 12 and 24 months, children develop a general (i.e., conceptual) ability to use spoons according to their canonical function effectively. Another study with implications for understanding the development of numerical skills is that of Scheuer and Sinclair (2009). They reported that, from 21 months, a girl began to use the same linguistic-numeric symbol (e.g., “one”) to designate identical and dissimilar exemplars of the same category (e.g., “one, one, one” to refer to three tiles or three identical T-shirts; “one, one” to refer to two different replica elephants). However, the girl did not extend this symbol to exemplars from other categories that were present at the time of categorising. Finally, Mervis (1985) noticed that, at 9 months, her son already knew how to blow in a canonical fashion a horn with which he interacted daily. She decided to explore whether or not this behaviour was general by providing the child with other household objects to play with. The results showed that the child also used his knowledge of the canonical use of the horn when interacting with a plastic bucket and an ice cream cone, thus suggesting the cognitive reality of a preverbal categorical representation.

## Discussion: methodological limitations

All tasks and procedures have provided valuable data to broaden our understanding of early conceptual development. However, there are methodological limitations that are worth briefly describing, both to consider the consequences of study conclusions with some caution and to suggest possible future avenues of research in the area.

**Fig. 3:**
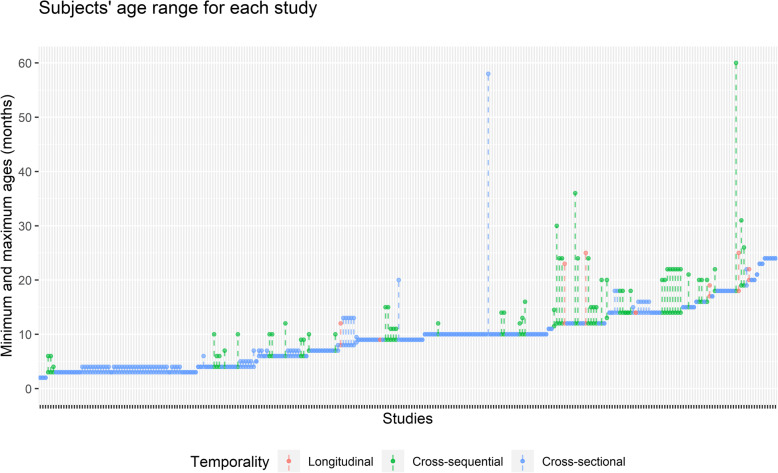
Subjects’ age range for each study

It is striking that almost all studies on conceptual development are cross-sectional or cross-sequential (96.8%), with *few studies being longitudinal* (3.2%) (see Fig. 3). It is not that these studies are not useful. However, there are aspects of cognitive development that can only be understood through longitudinal designs (Hoppe-Graff, 1989; Müller & Giesbrecht, 2008), such as the characteristics of intra-individual change and its causes, or the patterns of development associated with individual differences (Nesselroade & Baltes, 1979). While it is true that “effective longitudinal research on individual development must deal with substantive, far-reaching methodological and research strategy problems” and that “few areas of research are so full of traps as development research” (Magnusson, 1989, p. vii), it is also true that obtaining repeated intra-individual measures brings unparalleled security to cognitive development research (Grimm et al., 2017).

Similarly, most of the studies involve *experiments* (97.1%) and adopt a *quantitative approach* to data analysis (98.6%). Relegating qualitative information to the background is more than a research strategy: in the epistemological level, it assumes that phenomena can be explained by translating them into the language of numbers and applying to them a statistical treatment (Tuli, 2011). Also, the fact that most studies have been conducted in laboratory settings has some unintended consequences for research (Lécuyer & Rovira, 1999). One of them is that experimental control significantly reduces the degree of external validity of the designs. The critical point is that researchers can provide very accurate information about what happened in the laboratory after the manipulation of each independent variable, but they cannot guarantee that what they say applies to the real world (which is supposed to be the *explanandum* that makes research reasonable).

There are several examples of asymmetries between what happens in the laboratory and what happens in the everyday world. The first of them relates to the *referents of children's categorical representations*: what do children categorise in everyday life? Studies have used a variety of stimuli, ranging from abstract patterns of movement or images to replicas and non-objects specially constructed for experiments. These are mostly abstract and artificial stimuli (Sakharov, 1990) whose structure is not analogous to real-world stimuli (Anderson & Prawat, 1983). Rakison and Butterworth (1998b), for instance, recognise (though do not share) that it is possible to think that “a ‘cow with wheels’ is simply no longer a cow” (p. 58). This dissociation between the laboratory and the real world is often a deliberate investigative strategy to achieve greater control of variables or to isolate the effect of a particular variable (e.g., the influence of form vs. function of objects on categorisation, or differences in categorisation of objects with more or less obvious perceptual properties; Corrigan & Schommer, 1984; Perone & Oakes, 2006; Strauss, 1979; Welder & Graham, 2006). However, the cost of achieving a higher degree of internal validity is, in this case, to bypass the objects to which children have access in everyday life (e.g., instruments and artefacts used during mealtime). This, in turn, reduces the degree of external validity of the designs and entails a first degree of denaturalisation of the object of study.

**Fig. 4:**
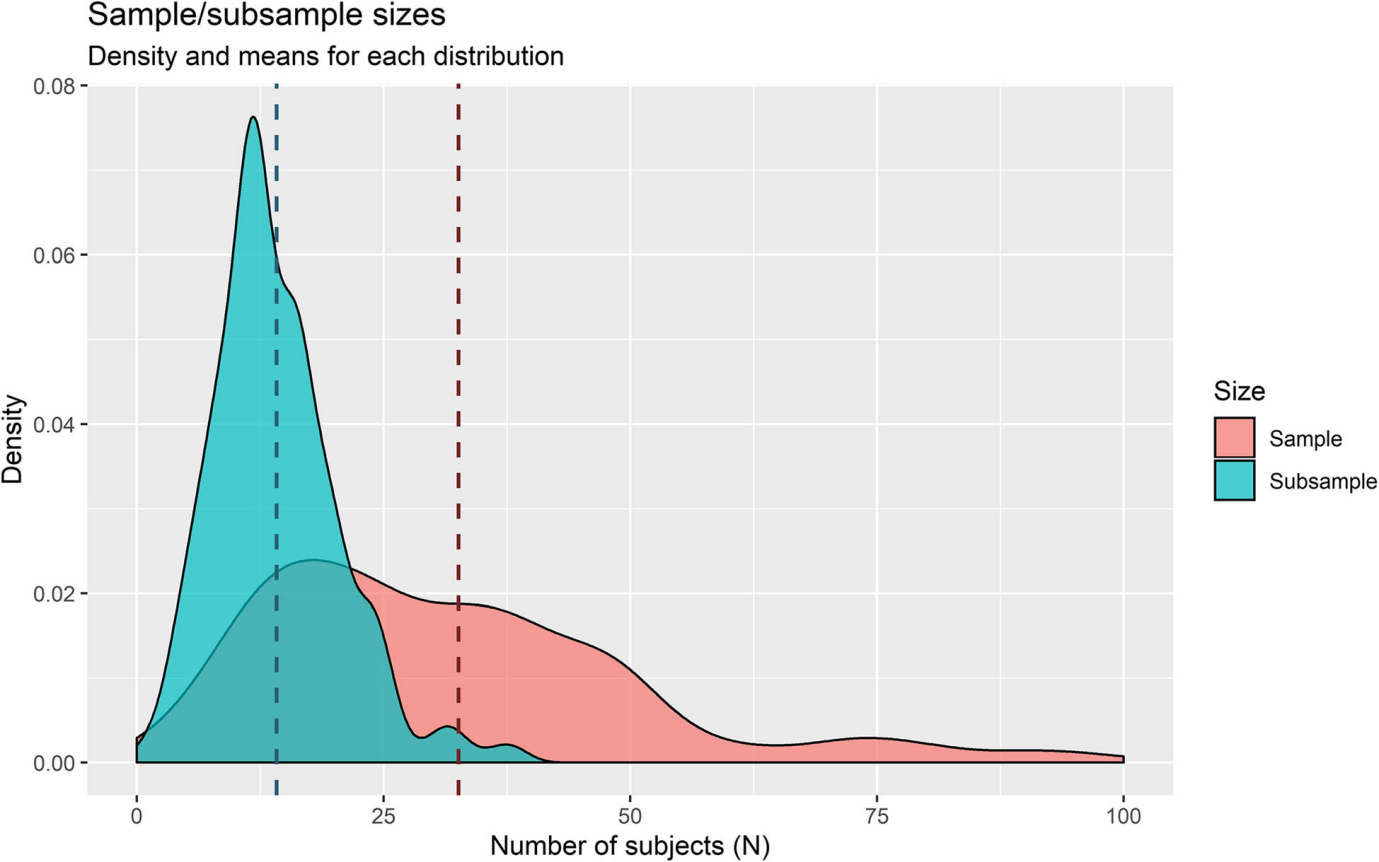
Sample/subsample sizes. Densities and means for each distribution

Another limitation concerns the *sample size of the studies*. One of the advantages usually attributed to laboratory tasks is that, as they are shorter and more controlled than other studies (e.g., descriptive longitudinal), they can involve larger samples. This is relevant because, according to the Central Limit Theorem (Kwak & Kim, 2017), if random samples of *n* observations are chosen from a population with mean *μ* and standard deviation *σ*, the larger *n* is, the closer the sample distribution of means will be to a normal distribution with mean *μ* and standard deviation $\sigma /\sqrt{n}$. Thus, a large *n* (greater than or equal to 30) (Salkind, 2012) allows the use of more accurate and reliable parametric statistics than non-parametric ones and, therefore, the generalisation of the results to the population of reference. However, in the studies we reviewed, total sample sizes only exceed 30 subjects in 50.17% of the studies (*M* = 32.56, *SD* = 21.52) and the number of subjects in the subsamples per experimental condition is only higher than or equal to 30 in 3.2% of the cases (*M* = 14.13, *SD* = 6.34) (see Fig. 4). This is not a minor issue. As others have noted, the use of small samples is a severe problem, as it undermines the statistical power of experimental explanatory studies by making the results neither meaningful nor reproducible, especially in the case of psychological and neuroscientific literature (e.g., Bakker et al., 2012; Button et al., 2013; Marszalek et al., 2011). In this way, it does not seem plausible to justify the realisation of experimental studies alluding to the advantages they could provide for the generalisation of the results^2^.

Consideration should also be given to the *relationship between the stimuli and the categories* whose formation is to be assessed. The question is whether a differential response to drawings and images (26.6% of the studies) or replicas (23.5%) points to the existence of the concept of the objects these stimuli represent. Although some argue that it does (McDonough & Mandler, 1998, p. 231), there is also evidence that this would not be the case (Arterberry & Bornstein, 2012; Arterberry et al., 2013; DeLoache, 2000; Flavell et al., 1990). The difficulty lies in the symbolic character of images and replicas: “before one can understand or use any symbol, one must first realise that it is a symbol, that is, that it stands for or represents something other than itself” (DeLoache, 1991, p. 736). This would seem to suggest that for the formation of the first concepts it is necessary to have first developed symbolic skills and dual representations, which is anti-intuitive and incongruent with much of the academic literature on the subject (for a review of symbolic development see Rodríguez et al., 2014).

Third, we should not forget that categorisation takes place within the framework of *organised everyday activities* and not in a vacuum (e.g., Nelson, 1985; Mandler et al., 1987). These everyday activities are not equivalent to experimental tasks. In preference and habituation experiments, for example, the test trials are successive and extraordinarily brief and, once completed, stimuli disappear. This contrasts with the time children spend interacting with objects in everyday life and with the stimuli availability of the real world (Corrigan & Schommer, 1984).

On the other hand, there is evidence that children under 24 months have severe difficulties in retaining the conceptual categories learned during experimental tasks for more than a few minutes (Merriman et al., 1997; Rose et al., 2001; Sherman, 1985; see also Oakes & Kovack-Lesh, 2013). This *sudden amnesia* (Hayne et al., 1987) contrasts with what happens in adult cognition, where categories amplify action possibilities (Tulving, 1983). In addition, these oblivions do not satisfy the *temporal stability requirement* that Schlesinger (1982) proposed to distinguish children conceptual behaviours. In this way, the pragmatic usefulness of the concepts reported in experiments is questionable: Why would children have developed such volatile conceptual representations? From a functional perspective, Nelson (1989) has argued that memory is a by-product of everyday life and that “remembering the past has value insofar as it serves action in the present or future. Thus what is remembered should be that that enables the individual to carry out activities, to predict and to plan” (Nelson, 1989, p. 144).

**Fig. 5:**
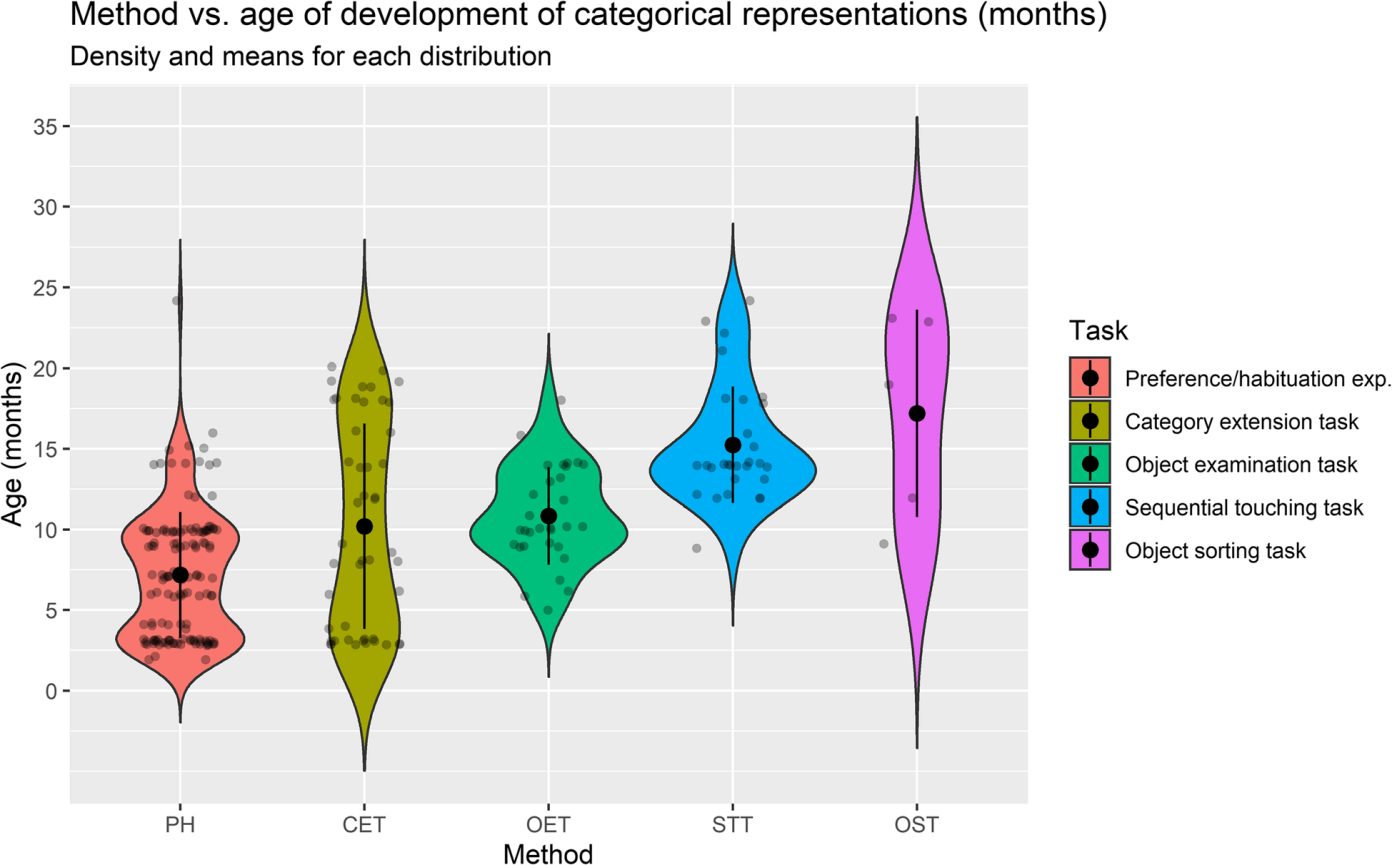
Method vs age of development of categorical representations (months). Density and means for each distribution

**Fig. 6:**
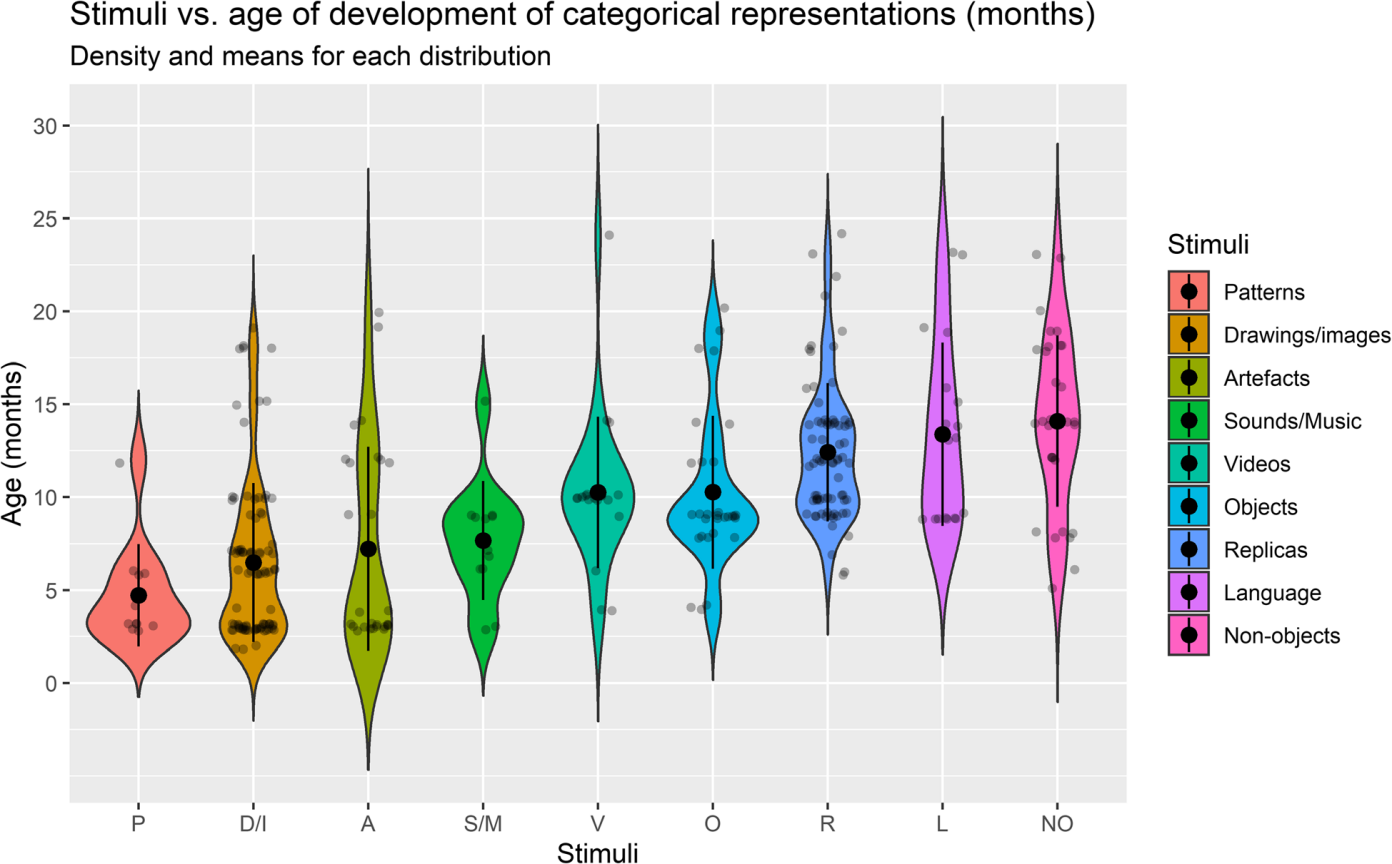
Stimuli vs age of development of categorical representations (months). Density and means for each distribution

This suggests that an ecological study of categorisation should consider the challenges, interests and goals that children face in their everyday contexts. At the same time, one must consider the *great variety of tasks and stimuli* used in studies, perhaps inherited from the lack of clear criteria on what it means to speak of categorisation or early conceptual formation (Oakes et al., 1991). This procedural breadth adds up to the great variety of concepts being investigated (e.g., belonging to different domains) and their multiple levels of complexity (e.g., basic, superordinate, global). Overall, these factors make results highly entropic and make it impossible to reach an agreement as to when categorical representation abilities would develop (see Figs. 5 and 6). It should be noted that the disparateness of concepts under research is due, at least in part, to differences between the theories that underlie empirical undertakings and between the hypotheses that these theories seek to validate. Thus, some of the theoretical debates concern, for instance, whether in early development (i) concepts are prototypical representations (e.g., Rosch, 1998) or represent sets of exemplars (e.g., Hayne et al., 1987), (ii) whether concepts are based on perceptual (e.g., Fagan, 1976; Welder & Graham, 2006) or functional object attributes (e.g., Booth et al., 2010; Nelson, 1973), (iii) whether basic-level (e.g., Mervis & Crisafi, 1982) or global-level categories (e.g., Mandler & McDonough, 1993; Rostad et al., 2012; Quinn, 2004) are developed first, (iv) whether the structure of concepts resembles that of a complex scientific theory (e.g., Gopnik, 1984) or is formed by everyday particular encounter with objects (e.g., Tomasello, 1999), (v) whether conceptual knowledge is organised in partonomies rather than taxonomies (e.g., Tversky & Hemenway, 1984) and (vi) whether language is essential to concept formation (e.g., Ferry et al., 2010; Xu, 1998)^3^.

This links to the debate on the value of *empirical indicators considered in each methodological perspective*. Visual preference methods, for example, exhibit two problems. On the one hand, it is not clear that an increase in gaze fixation times reflects categorical knowledge. It could be, instead, the result of basic attentional processes not accessible to consciousness (Bonatti et al., 2002). On the other hand, in these experiments, the subject is a passive spectator only allowed to respond through his gaze. In addition to being uncomfortable for children (Ruff, 1978), this procedure also contradicts everyday experience, where action is of paramount importance (Babska, 1965). Those who have researched through object examination tasks emphasise, precisely, the advantage that this task supposes in allowing the child to interact with objects (e.g., Mandler et al., 1987, p. 352). However, in these tasks, action is restricted, due to the control measures that the laboratory experimentation requires.

Another ubiquitous bias among studies is that they consider categorisation as an *individual achievement*. Children would form categories through direct contact with the stimuli proposed by the researchers. Adult influence is dismissed, because it is assumed that adults’ cognitive capacities have already been “obscured by layers of acquired knowledge and idiosyncratic processing strategies” (Quinn et al., 1993), by the cultural niches in which they live. However, there are two reasons to doubt the individualistic stance. First, from the beginning of life, children are part of early triadic interactions in which adults act as ambassadors for the material world. If children manage to construct a conceptual system like that of adults, this is not due to chance, but to a large number of semiotic communicative mediators that adults incorporate in interactive situations to share meanings (Alessandroni et al., 2019; Moreno-Núñez et al., 2017; see also Luce & Callanan 2010 on parents' object labeling). Second, if concepts are powerful cognitive tools for communication, it is unlikely that subjects will develop autonomous conceptual systems, as this would make interpersonal understanding impossible. Rather, what should be explained is how the intersubjective cognitive agreements that constitute the *common ground* of categorisation and communication through concepts are constructed (see Rodríguez, 2015; Waxman et al., 2016).

Finally, most studies assume that concepts are *perceptual distillates*. Categories would result from comparing and abstracting physical properties of objects (e.g., Bomba & Siqueland 1983; Casasola & Ahn 2018; Quinn & Johnson 2000; Ruba et al., 2017; Stavans & Baillargeon 2018; Younger & Fearing 1999, 2000). However, this prospect raises hard problems at the epistemological level (for an in-depth discussion, see Alessandroni, 2020; Alessandroni & Rodríguez, in press). There may be other bases for conceptual formation, such as *object function* (Barsalou et al., 2018; Nelson, 1973, 1983; Oakes, 2008). Although some studies assume this functional perspective, there is no agreement on the definition of “function”. Sometimes, “function” refers to everything we can do with objects, that is, to the set of action possibilities that objects enable. This definition correlates to the concept of *affordance* proposed by Gibson (1979/2015). In opposition, the perspective of the *pragmatics of the object* (Rodríguez et al., 2018; Rodríguez & Moro, 1999) has proven that among this set of possible actions some are culturally privileged and are primarily communicated in interactive situations: the *canonical functions of objects*. These are not the actions that *can*, but those that *must*, be carried out with objects in a given sociocultural and historical context (e.g., cups should be used for drinking). This type of knowledge is indispensable for children, as it provides them with a criterion of public objectivity. Thanks to it, children become cultural agents who align their actions and objectives with social norms. In any case, the distinction between these two levels of analysis (Borghi, 2005) seems crucial for the study of categorisation. Possessing the concept of fork involves not only information about forks’ physical characteristics and everything that could be done with them, but also information about how one should act with forks in our cultural environments. The lack of canonical knowledge would lead children to a state of permanent uncertainty about others’ actions and a state of paralysis concerning their own action.

## Conclusions

In recent years, a good deal of evidence has accumulated on the early development of categorisation skills and conceptual thinking. The results suggest that from 2 months of age, children can form categorical representations in a wide variety of domains and levels of abstraction. The experiments of preference and habituation, clearly dominant in the research scene, as well as the other tasks and procedures, have challenged the traditional conception of the baby as a subject trapped in the dimension of the singular. From the first months of life, long before the development of articulated language, children can think in general terms.

However, the studies exhibit a number of methodological limitations relating to (i) the temporality of research designs, (ii) the stimuli validity, (iii) the sample and subsample sizes; (iv) the data analysis approach, (v) the mismatch between the nature of tasks and children's real-world experience, (vi) the empirical indicators that are considered as sufficient to speak of categorical representation and (vii) some epistemological and theoretical assumptions about the bases of categorisation.

Wishing to unravel the way children conceptualise the real world by investigating their behaviour in artificial worlds is, clearly, of no investigative relevance (Kemler Nelson, 1990, p. 606). In that sense, our review points to the urgency of proposing new paradigms for the study of conceptual development. First, there is a strong need to investigate the dynamics of conceptual development as it takes place in everyday life, in the context of structured activities, considering not only the perceptual properties of stimuli but also their functional aspects. In this regard, we briefly highlighted the practical importance of knowledge about the canonical function of objects: it allows us to orientate in the world and to plan our action. There is general agreement that these two are preconditions for talking about concepts. Besides, knowledge of canonical object functions provides a way to explain the communicative power that categorical representations acquire once they have been formed, appealing to cultural processes of meaning and rule construction. If, as Nelson (1973, 1983) and Mervis (1985) argue, this type of knowledge is fundamental for the formation of the *intensional core* of object concepts in early childhood, it would be worthwhile to investigate it with greater precision. To achieve this goal, it would be desirable to carry out longitudinal studies in ecological contexts such as children’s home and Early Childhood Education settings (Gruber et al., 2015; Murphy, 2002). Perhaps, these are the two most critical everyday contexts for babies, as this is where they tend to spend the most time. Finally, we believe that to advance research in this area, we should consider larger units of analysis that reflect the intersubjective networks through which children categorise the world. For all these reasons, a non-reductive model of children’s conceptual development requires a serious investigation of the structural and dynamic characteristics of the communicative and semiotic mediation processes that adults bring into play during the processes of children's conceptual formation, as well as their relationships with the pragmatic and sociocultural complexity of the material world.

## Data Availability

The datasets used and/or analysed during the current study are available from the corresponding author on reasonable request.

## Footnotes

1 It goes without saying that this definition poses problems due to ontological and epistemological commitments. There are other definitions of what a *concept* is, specific to non-orthodox theoretical stances and even disciplines (e.g., a concept in psychology is not the same as in the philosophy of the mind). This not only happens with the term “concept,” but affects many other key terms, such as “representation”, “intentionality”, “intelligence” or “mind”. We have considered here a generic definition on which there would be consensus among most psychologists (see Nelson 2011).

2 Indubitably, whether they are cross-sectional or have small samples is not a problem for *exploratory* studies that do not intend to lead to conclusive or generalisable results. However, it becomes a problem as soon as studies employ experimental designs that seek to measure the effect of an independent variable on a dependent variable and in which data analysis makes use of parametric tests (e.g., ANOVA). This is because parametric tests, as hypothesis testing procedures, are based on assumptions about the distribution of the underlying population distribution (e.g., the data are approximately normally distributed). Since in parametric tests a sample statistic is obtained to estimate a population parameter and the vast majority of studies that we reviewed make use of this type of tests, it is clear that they are intended to be *explanatory* rather than exploratory. We would like to thank an anonymous reviewer for emphasising this point.

3 Due to the methodological nature of this review, we do not explore here the practical consequences of different theoretical frameworks (for a theoretical analysis, see Alessandroni, 2020; Alessandroni & Rodriguez, *in press*).
